# The Impact of Telemedicine on Human Immunodeficiency Virus (HIV)-Related Clinical Outcomes During the COVID-19 Pandemic

**DOI:** 10.1007/s10461-024-04342-x

**Published:** 2024-04-25

**Authors:** Avery Lin Cox, Daniel Tsang, Lisa A. Spacek, Constantine Daskalakis, Dagan Coppock

**Affiliations:** Sidney Kimmel Medical College, Department of Medicine, Division of Infectious Diseases, Thomas Jefferson University, Philadelphia, PA United States of America

**Keywords:** HIV, Telemedicine, Telehealth, Adherence

## Abstract

The coronavirus disease of 2019 (COVID-19) pandemic exacerbated barriers to care for people living with human immunodeficiency virus (HIV) (PLWH). The quick uptake of telemedicine in the outpatient setting provided promise for care continuity. In this study, we compared appointment and laboratory no-show rates in an urban outpatient HIV clinic during three time periods: (1) Pre-COVID-19: 9/15/2019–3/14/2020 (predominately in-person), (2) “Early” COVID-19: 3/15/2020-9/14/2020 (predominately telemedicine), and (3) “Later” COVID-19: 9/15/2020-3/14/2021 (mixed in-person/telemedicine). Multivariable logistic regression models evaluated the two study hypotheses: (i) equivalence of Period 2 with Period 1 and of Period 3 with Period 1 and (ii) improved outcomes with telemedicine over in-person visits. No-show rates were 1% in Period 1, 4% in Period 2, and 18% in Period 3. Compared to the pre-pandemic period, individuals had a higher rate of appointment no-shows during Period 2 [OR (90% CI): 7.67 (2.68, 21.93)] and 3 [OR (90% CI): 30.91 (12.83 to 75.06). During the total study period, those with telemedicine appointments were less likely to no-show than those with in-person appointments [OR (95% CI): 0.36 (0.16–0.80), *p* = 0.012]. There was no statistical difference between telemedicine and in-person appointments for laboratory completion rates. Our study failed to prove that no-show rates before and during the pandemic were similar; in fact, no-show rates were higher during both the early and later pandemic. Overall, telemedicine was associated with lower no-show rates compared to in-person appointments. In future pandemics, telemedicine may be a valuable component to maintain care in PLWH.

**Supplementary Information:**

The online version contains supplementary material available at 10.1007/s10461-024-04342-x.

## Introduction

Despite the proven effectiveness of antiretroviral therapy (ART) for suppressing human immunodeficiency virus (HIV), people living with HIV (PLWH) may experience numerous barriers to care, which can, in turn, lead to an increase in treatment no-shows [1–3]. With the arrival of the coronavirus disease of 2019 (COVID-19) pandemic in the United States, PLWH faced additional challenges in accessing care [4, 5]. To ameliorate these early-pandemic challenges, the Infectious Disease Society of America and the HIV Medicine Association recommended the use of telemedicine in HIV clinics [6].

At the onset of the pandemic, the Thomas Jefferson University (TJU) HIV clinic shifted from an all in-person model to one that heavily relied upon telemedicine. Our hope was that telemedicine would allow the clinic to maintain a level of care like that of the pre-pandemic era. At the time, telemedicine was a novel entity and little was known about its impact on adherence. While a few studies have explored its effect on adherence, results varied. One study found that appointment adherence increased during the pandemic due to telehealth [7]. Our study aimed not only to address appointment and laboratory adherence, but also to examine the time period when both telemedicine and in-person visits were available to patients.

While some studies showed improvement in adherence with telehealth, others noted difficulty due to transportation barriers to care and increased rates of mental health challenges, such as depression [3, 7]. In this study, we hypothesized that pre- and intra-pandemic time periods would have similar rates of appointment no-shows and laboratory completion. We further hypothesized that these clinical outcomes would be superior for telemedicine appointments compared to in-person visits.

## Methods

All individuals with appointments scheduled at the TJU outpatient HIV clinic in Philadelphia, Pennsylvania during the study period were included in the study. There were no other demographic inclusion or exclusion criteria. The study included 3 discrete six-month periods: (1) Pre-COVID-19: 9/15/2019–3/14/2020 (Period 1, predominantly in-person), (2) “Early” COVID-19: 3/15/2020-9/14/2020 (Period 2, predominantly telemedicine), and (3) “Later” COVID-19: 9/15/2020-3/14/2021 (Period 3, mixed in-person/telemedicine). These time periods served as natural six-month inflection points when appointment types changed across the United States healthcare system. Variables collected were available as structured data within the electronic health record (EHR). Patient data was extracted from the EHR by TJU Information Science based upon appointment schedule. HIV clinic appointments were defined as appointments at the TJU HIV clinic location with one of the specific HIV providers.

Outcome definitions were based on index appointments. Index appointments were defined as the first scheduled appointment within a given time period; subsequent visits from patients in a given time period were not included in the analysis. The appointment no-show rate was calculated as the number of missed index appointments divided by the total number of index appointments. Re-scheduled appointments were not included in the analysis. To establish a completion rate definition, we assumed that HIV viral load, cluster of differentiation 4 (CD4), and sexually-transmitted infection (STI) testing should occur at least every 6 months based upon Ryan White program requirements [8]. Therefore, the laboratory completion rate was calculated as the number of a given laboratory test completed within 6 months of index appointments divided by the total number of index appointments. Laboratory tests that were evaluated included CD4, HIV viral load, gonorrhea nucleic acid amplification test (NAAT) (oropharyngeal, urinary, and rectal sites collected), chlamydia NAAT (oropharyngeal, urinary, and rectal sites collected), and syphilis test (rapid plasma reagin, RPR). Additionally, the percentage of detectable HIV viral loads was calculated by time period and appointment type. A detectable viral load was defined as > 200 copies/mL based upon Centers for Disease Control (CDC) definition [9].

Multivariable logistic regression models that controlled for patient characteristics including sex, age, race, and insurance, as well as Ryan White registry, and new/established patient status evaluated the 2 study hypotheses: (i) equivalence of Period 2 with Period 1 and of Period 3 with Period 1 and (ii) improved outcomes with telemedicine over in-person visits (Supplementary Table 1). These hypotheses were assessed separately for each study outcome. Equivalence of the time periods was defined on an odds ratio (OR) scale, with margins specified a priori, based on clinical judgment. For appointment no-shows (expected rate ~ 5%), equivalence was prespecified as OR between 0.5 and 2 (approximately corresponding to rates from 2.6 to 9.5%). For CD4 and viral load testing completion (expected rate ~ 60%), equivalence was set as 0.67 to 1.50 (rates from 50 to 69%), while for RPR and gonorrhea/chlamydia testing (expected rate 25%), equivalence was set as 0.75 to 1.33 (rates from 20 to 31%). The equivalence hypothesis was assessed with two one-sided tests (TOST approach) and 90% confidence intervals (CIs) were computed; all other results are presented with the usual 95% CIs. All analyses used the Generalized Estimating Equations approach with the robust variance to account for the fact that some patients contributed multiple observations (appointments) across the time periods.

## Results

Nine hundred fifteen scheduled office visits for 515 unique patients were evaluated. There were 250 patients with appointments in one time period, 241 with appointments in two time periods, and 61 with appointments in all three time periods. In Periods 1, 2, and 3, 376, 316, and 223 appointments were scheduled, respectively. Demographic data were similar between all three time periods (Table 1). In Period 1, there were no scheduled telemedicine visits, while in Periods 2 and 3, 218 (69%) and 67 (30%) were telemedicine visits, respectively.

**Table 1:** Demographic characteristics of those seen for appointments at the Thomas Jefferson University HIV Clinic. Total number of unique patients attending clinic, *N* = 515

Period	1: Pre-COVID	2: Early COVID	3: Late COVID
Time Period	9/15/19 − 3/14/20	3/15/20 − 9/14/20	9/15/20 − 3/14/21
Length (days)	181	183	180
Appointments	*N* = 376	*N* = 316	*N* = 223
**Variable**			
Sex, *n* (%)			
Female	83 (22)	56 (18)	46 (21)
Male	293 (78)	260 (82)	177 (79)
Age, *n* (%)			
<40	136 (36)	129 (41)	81 (36)
40–49	91 (24)	77 (24)	51 (23)
50+	149 (40)	110 (35)	91 (41)
Race, *n* (%)			
White	107 (28)	87 (28)	56 (25)
African American	225 (60)	192 (61)	146 (65)
Hispanic	28 (7)	26 (8)	16 (7)
Other	16 (4)	11 (3)	5 (2)
Insurance,* *n* (%)			
Private	252 (69)	226 (73)	152 (71)
Medicaid	29 (8)	25 (8)	19 (9)
Medicare	85 (23)	58 (19)	44 (20)
Ryan White registry, *n* (%)	257 (68)	218 (69)	164 (74)
New patient, *n* (%)	39 (10)	36 (11)	22 (10)
Telemedicine visit, *n* (%)	0 (0)	218 (69)	67 (30)
Provider, *n* (%)			
1	2 (1)	18 (6)	18 (8)
2	210 (56)	149 (47)	95 (43)
3	0 (0)	1 (0)	0 (0)
4	164 (44)	148 (47)	110 (49)

(*) Data missing for 25 appointments

### Comparison of Periods 2 and 3 vs. Period 1: No-Show Rates

No-show rates were 1% in Period 1, 4% in Period 2, and 18% in Period 3 (Table 2). In the multivariable model, which included time period and type of appointment (in-person or telemedicine), as well as patient characteristics, the no-show rate in Periods 2 and 3 remained substantially higher than in Period 1 (odds ratios, OR = 7.67 and 30.91, respectively; equivalence *p* = 0.982 and 0.999, respectively (Table 2). Based on these findings, the equivalence of the intra-COVID-19 Periods 2 and 3 vs. the pre-COVID Period 1 was not established (Table 2).

**Table 2:** Multivariable results for appointment no-shows (*N* = 915)

	Scheduled Appointments	No-show: *n* (%)		OR	(CI)	*P*
**Hypothesis:** **Equivalence** **Total**	915	58	(6%)			
**Period**						
1	376	4	(1%)	1.00	Ref	
2	316	13	(4%)	7.67	(2.68, 21.93)	0.982*
3	223	41	(18%)	30.91	(12.83, 75.06)	0.999*
**Hypothesis**: **Difference** **Visit type**						
In person	630	45	(7%)	1.00	Ref	
Telemedicine	285	13	(5%)	0.36	(0.16, 0.80)	0.012

OR: odds ratio. CI: confidence interval (90% for Period and 95% for visit type). Results are adjusted for patient sex, age, race, and insurance, as well as Ryan White registry, and new/established patient status

(*) *P*-value for equivalence, with OR margin [0.5, 2.0] (*p* < 0.05 indicates equivalence established)

### Comparison of Periods 2 and 3 vs. Period 1: Laboratory Completion Rates.

Comparisons of Periods 2 and 3 with Period 1 (equivalence hypotheses) on lab testing completion are summarized in Table 3. Gonorrhea and chlamydia were tested for either both or neither in 100% of cases, so their analysis was performed as a single outcome. For all labs, completion rates were very similar across the three periods, but equivalence was formally established only for Periods 1 and 3 on CD4 (OR = 0.95, equivalence *p* = 0.024) and viral load (OR = 1.05, equivalence *p* = 0.027). The percentage of detectable viral loads were 10% in Period 1, 6% in Period 2, and 9% in Period 3 (Supplementary Table 2).

**Table 3:** Multivariable results for equivalence of intra-COVID Periods 2 and 3 with pre-COVID Period 1 on lab testing adherence (*N* = 915)

Lab		Scheduled Appointments	Labs completed: *n* (%)		OR	(90% CI)	P*
CD4	**Total**	915	538	(59%)			
	**Period**						
	1	376	223	(59%)	1.00	Ref	
	2	316	183	(58%)	0.91	(0.65, 1.28)	0.065
	3	223	132	(59%)	0.96	(0.71, 1.30)	0.024
Viral	**Total**	915	551	(60%)			
Load	**Period**						
	1	376	224	(60%)	1.00	Ref	
	2	316	188	(59%)	0.89	(0.63, 1.25)	0.088
	3	223	139	(62%)	1.05	(0.77, 1.42)	0.027
RPR	**Total**	915	202	(22%)			
	**Period**						
	1	376	86	(23%)	1.00	Ref	
	2	316	70	(22%)	0.79	(0.52, 1.20)	0.426
	3	223	46	(21%)	0.79	(0.55, 1.15)	0.403
Gonor/	**Total**	915	300	(33%)			
Chlam	**Period**						
	1	376	121	(32%)	1.00	Ref	
	2	316	105	(33%)	1.22	(0.83, 1.78)	0.349
	3	223	74	(33%)	1.12	(0.80, 1.56)	0.192

OR: odds ratio. 90% CI: 90% confidence interval. Results are adjusted for patient sex, age, race, and insurance, as well as Ryan White registry, and new/established patient status

(*) *P*-value for equivalence, with OR margin [0.67, 1,50] for CD4 and viral load, and [0.75, 1.33] for RPR and gonorrhea/chlamydia (*P* < 0.05 indicates equivalence established)

### Comparison of Telemedicine vs. In-person Visits: No-Show Rates.

Patients with telemedicine appointments were less likely to no-show for an appointment than those with in-person appointments (OR = 0.36, *p* = 0.012, Table 2). In further analyses that included the interaction between time period and visit type, a benefit of telemedicine over in-person visits was seen in Period 2 (2% vs. 9%, OR = 0.15, *p* = 0.003). This was not significant in Period 3 (13% vs. 21%, OR = 0.57, *p* = 0.181; interaction *p* = 0.065).

### Comparison of Telemedicine vs. In-person Visits: Laboratory Completion Rates.

Comparisons of telemedicine and in-person visits on lab testing completion are summarized in Table 4. Laboratory completion rates were generally similar for the two visit types. There was no statistical difference between telemedicine and in-person appointments. Furthermore, there was no suggestion that the difference varied across periods (p for interaction between period and visit type > 0.25 for all four labs). The percentage of detectable viral loads were 9% for in-person visits and 7% for telemedicine visits.

**Table 4:** Multivariable results for difference between telemedicine and in-person visits on lab testing adherence (*N* = 915)

Lab		Scheduled Appointments	Labs completed: n (%)		OR	(95% CI)	*P*
CD4	**Total**	915	538	(59%)			
	**Visit type**						
	In person	630	377	(60%)	1.00	Ref	
	Telemedicine	285	161	(56%)	1.02	(0.68, 1.52)	0.925
Viral	**Total**	915	551	(60%)			
Load	**Visit type**						
	In person	630	380	(60%)	1.00	Ref	
	Telemedicine	285	171	(60%)	1.15	(0.77, 1.71)	0.497
RPR	**Total**	915	202	(22%)			
	**Visit type**						
	In person	630	138	(22%)	1.00	Ref	
	Telemedicine	285	64	(22%)	1.23	(0.77, 1.98)	0.382
Gonor/	**Total**	915	300	(33%)			
Chlam	**Visit type**						
	In person	630	216	(34%)	1.00	Ref	
	Telemedicine	285	84	(29%)	0.68	(0.43, 1.08)	0.104

OR: odds ratio. 95% CI: 95% confidence interval. Results are adjusted for patient sex, age, race, and insurance, as well as Ryan White registry, and new/established patient status

## Discussion

Prior to the COVID-19 pandemic, telehealth technology appeared to be a promising tool for improving the care of PLWH [10, 11]. Recent data bears this out, indicating that telemedicine in the early pandemic led to improved clinical outcomes in this population [7, 12]. However, our study did not demonstrate equivalence between pre- and intra-pandemic time periods regarding appointment no-show rates, after accounting for the increased use of telemedicine during the pandemic and patient characteristics. In fact, our data demonstrate that no-show rates increased substantially during the pandemic.

Furthermore, over time, the absolute number of scheduled appointments dropped drastically from period to period. This is consistent with available data. As of June 2020, a study from the CDC demonstrated that 31.5% of patients with chronic conditions had postponed care due to concerns over COVID-19 [13]. A study of attitudes during the early pandemic suggested that fear of exposure and financial hardship experienced as a result of the pandemic contributed to postponed care [14]. Neither of these studies examined whether overall postponement differently impacted attended versus scheduled appointments. In the case of our study, both the rate of appointment attendance as well as the absolute number of scheduled appointments decreased. These decreases may be explained by care postponement or appointment rescheduling, though there may be socioeconomic explanations. Of note, the TJU HIV clinic was closed to patients to limit in-person contact from mid-March to the first week of June 2020. In the first half of Period 2, patients could only complete visits via telemedicine. As the TJU HIV clinic largely consists of Ryan White eligible patients, eligibility is based on the federal poverty level. For individuals with socioeconomic disparities, there is evidence to suggest that telehealth may paradoxically worsen access to care [15].

Our study suggests that telemedicine was associated with lower no-show rates than in-person visits, controlling for time periods and other covariates. Telemedicine may decrease the burden of travel to appointments and increase appointment scheduling flexibility [16]. However, the digital literacy and access to technology required to complete a telemedicine visit may be obstacles to care for some patients [16]. This may account for the findings in our study, which suggest that, despite the association with lower no-show rates, telemedicine was not associated with an equivalence in outcomes between pre- and intra-pandemic time periods.

Laboratory testing rates were very similar across all 3 periods, although formal equivalence was only established for CD4 and viral load for Period 3 vs. Period 1. For HIV viral load values, there was no meaningful clinical difference in rates of viral load detectability between time periods or between visit types. Though our study did not examine the mechanisms behind these findings, other studies postulate that lower laboratory testing rates during the pandemic may have been exacerbated by preexisting systemic barriers to healthcare utilization, hesitancy to interact in person [17], or, for individuals who did complete in-person visits, a lack of on-site testing [18], as is the case in our clinic. One study shows that many patients avoided routine medical care for fear of contracting COVID-19 [13]. The confusion from rapidly changing public health guidelines may have exacerbated disparities in healthcare access [13].

Our study had limitations, but these limitations provide opportunities for potential avenues for future research. One of the main limitations was the presence of several unmeasured variables that likely changed over time. We did not account for the shifting nature of the virus—the predominant strain at a given time and the relative virulence of those strains—or the shifting availability of interventions, including available preventative measures, such as vaccination and prophylactic monoclonal antibodies, and medications used for treatment. These changes might have affected our patients’ perceptions and, in turn, influenced appointment and laboratory no-show rates. Another study limitation was our focus on global appointment adherence as a unit of study as opposed to the tracking of adherence on a patient-by-patient level. And, we did not account for rescheduled visits, which may have affected clinical outcomes. We were also limited in our assumptions regarding our laboratory completion measure, which may not reflect true testing variability. Furthermore, our choice of equivalence margins, were relatively wide. In absolute terms, the margins for STI screening were slightly wider due to greater variability in completing STI tests compared to CD4 and HIV viral load tests. Future research efforts will focus on a more granular exploration of longitudinal test completion as impacted by telemedicine.

In summary, our clinic continued to provide access to care for PLWH during the tumultuous early days of the COVID-19 pandemic. While our study suggested a benefit of telemedicine versus in-person visits for no-show rates, telemedicine was not a panacea for all the challenges faced throughout the pandemic. When future pandemics arise, further strategies will need to be developed to preserve clinical standards for PLWH.

### Electronic Supplementary Material

Below is the link to the electronic supplementary material.

Supplementary Material 1
