# Supplementary material for: The Impact of Telemedicine on Human Immunodeficiency Virus (HIV)-Related Clinical Outcomes During the COVID-19 Pandemic
Source: AIDS Behav. 2024 Apr 25;28(7):2438–43. doi: 10.1007/s10461-024-04342-x (PMC11199220; doi:10.1007/s10461-024-04342-x)
Supplement: Supplementary file 1 — Supplementary Material 1 [file 10461_2024_4342_MOESM1_ESM.docx]

**Appendix**

Supplementary Table 1. Outcome variable and the hypothesis testing concept.

| **ENDPOINT** | **Periods 2&3 vs. Period 1** | | **Telemedicine vs. In-person** | |
| --- | --- | --- | --- | --- |
|  | **Hypothesis: Equivalence** | | **Hypothesis: Difference** | |
|  | *Expected rate (all blocks)* | *Margins (OR scale)* | *Expected rate In-Person* | *Expected rate Telemedicine* |
| **Appointment no-show** | 5% | [0.5, 2.0] | 7-9% | 3-4% |
| **Testing completion** |  |  |  |  |
| **CD4** | 60% | [0.67, 1.50] | 55-60% | 60-65% |
| **VL** | 60% | [0.67, 1.50] | 55-60% | 60-65% |
| **RPR** | 25% | [0.75, 1.33) | 20-25% | 25-30% |
| **STIs** | 25% | [0.75, 1.33) | 20-25% | 25-30% |

Supplementary Table 2. Percentage of detectable HIV viral loads by time periods and appointment types.

| **VL** | **Total (N=551)** | | **Period 1 (N=224)** | | **Period 2 (N=188)** | | **Period 3 (N=139)** | | **In person (N=380)** | | **Telemed (N=171)** | | |
| --- | --- | --- | --- | --- | --- | --- | --- | --- | --- | --- | --- | --- | --- |
|  |  |  |  |  |  |  |  |  |  |  |  |  |  |
| **Detectable,*** n (%) | **47** | **9%** | 23 | 10% | 12 | 6% | 12 | 9% | 35 | 9% | 12 | 7% |  |
|  |  |  |  |  |  |  |  |  |  |  |  |  |  |
|  |  |  |  |  |  |  |  |  |  |  |  |  |  |
| **Detectable,**** n (%) | **107** | **19%** | 45 | 20% | 34 | 18% | 28 | 20% | 77 | 20% | 30 | 18% |  |

(*) Detectable defined as >200. (**) Detectable defined as >0 and/or greater than the assay's threshold of detectability.
